# Books and Authors

**Published:** 1910-12

**Authors:** 


### BOOKS AND AUTHORS

**Dawn of the Fourth Era in Surgery and Other Short Articles.** By **Robert T. Morris,** M.D., Professor of Surgery, New York Post-Graduate Medical School and Hospital. 12mo, of 145 pages. Philadelphia and London: W. B. Saunders Company, 1910. ($1.25 net.)

When we looked at the back of this book and saw "The Fourth Era in Surgery," we thought we had something to review; but when we read the title page and noted " Dawn of the Fourth Era in Surgery, and other short articles previously published," we began to feel doubt about attempting to display our talents (of course there is no doubt as to the propriety of using this last word) as a reviewer. When, finally, we read the preface all doubts were dispelled, and we knew our services were not required, that our splendid talents were dispensed with,—the author himself had reviewed the book better than could we, or anybody else.

But we cannot thus permit ourselves to be estopped from saying something about the book, or at least expressing our opinion in regard to it.

Some of these essays are full of import, while all are of value in teaching by epigrams truths that need rehearsing now and again, that their kernel may take deep root. The address at the annual meeting of the American Association of Obstetricians and Gynecologists at Detroit, September, 1907, entitled "Back to an Old Idea," in which the author discoursed upon the importance of conserving the natural immunity of the patient is one of the important articles in the book. Another is "Protective Appendicitis," which formed the subject of an address at the Budapest Congress in August, 1909. It is not easy to differentiate when all are so good. This is the sort of book to put in the pocket to read on short railway journeys. The expressions are original, the teachings are sound.

**Difficult Labor.** By **G. Ernest Herman,** M. B. Lond., Consulting Obstetric Physician to the London Hospital. 12 mo. pp. 559. Illustrated. New York: William Wood & Co., 1910. (Cloth, $2.50.)

The fifth edition of Herman's book follows along the lines adopted in previous revisions. His advice as to the management of difficult cases of labor is unusually sound and conservative. A chapter on puerperal eclampsia has been added to the new book in which the author advocates what he terms the morphia treatment. The summary of his treatment for eclampsia is quoted: "You are called to a patient who has had one or more fits; her urine is scanty and when boiled solid with albumin; she is insensible but restless. Give ½ gr. morphia with 1-60 gr. atropine by hypodermic injection. In half an hour, if the patient is not in deep sleep repeat the dose. If in half an hour after that the patient is still restless give ¼ gr. of morphia with 1-120 grain of atropine, and repeat every half hour until the patient is in a deep sleep."

---

**Transactions of the American Dermatological Association** at its thirty-third annual meeting held in Philadelphia, June 3-5, 1909. **Grover W. Wende,** M.D., secretary.

The meeting, of which this volume chronicles the proceedings was held at Philadelphia, June 3, 4 and 5, 1909, under the presidency of Dr. Thomas Caspar Gilchrist, of Baltimore. The book shows careful preparation and editing by the secretary, Dr. Grover William Wende, of Buffalo. One excellent feature of the transactions is that a list of publications of the members for the year preceding the meeting is printed at the end. A fine group of papers is published, most of them on technical subjects, and many of them are well illustrated. Dr. Wende, himself, publishes an article entitled "A Nodular Terminating in a Ring Erruption of the Skin," otherwise granuloma annulare. This interesting case is described in detail and a graphic illustration of the patient is published. The volume is of great interest to every one who practises dermatology.

**The Medical Record Visiting List or Physician's Diary for 1911.** New revised edition. New York: William Wood & Company, Medical Publishers. 1910.

In the December issue of this magazine for 1909 we published a review notice of this invaluable visiting memorandum book, and we are glad to receive it in season to publish this notice in a corresponding number of the JOURNAL this year. It is important that physicians should be supplied with their visiting lists for next year before 1911 sets in. In the 60 patients a week list before us, besides the space set apart for patients, we find a vast amount of valuable material useful in the sick room or at the bedside, such as a two years' calendar; estimation of the probable duration of pregnancy; approximate equivalents of temperature, weight, capacity, measure, and the like; maximum adult dose by the mouth in apothecaries' and decimal measures; drops in a fluid dram; solutions for subcutaneous injection; solutions in water for atomisation and inhalation; miscellaneous facts, emergencies, surgical antisepsis, disinfection, dentition, and table of signs; visiting list with special memoranda; consultation practice; obstetric engagements, and record of obstetrical practice; record of vaccination; register of deaths, nurses' addresses, addresses of patients and others, cash account, as well as other data and hints of value.

The prices of regular lists are, for sixty patients a week, with or without dates, handsomely selected red or black morocco binding, $1.50; for thirty patients a week with or without dates, same style, $1.25. Orders should be placed with promptitude, lest the edition become exhausted before the end of the present month.

**International Clinics.** A Quarterly of Illustrated Clinical Lectures and especially prepared articles on Treatment, Medicine, Surgery, Neurology, Pediatrics, Obstetrics, Gynecology, Orthopedics, Pathology, Dermatology, Ophthalmology, Otology, Rhinology, Laryngology, Hygiene, etc. Edited by **Henry W. Cattell**, M.D. Vol. III, twentieth series. Philadelphia and London: J. B. Lippincott Co. 1910. (Cloth, $2.00.)

This volume contains thirty-two articles distributed among the important subheads of medicine—namely, diagnosis, treatment, teeth and oral cavity, gynecology, medicine, and surgery, with a miscellaneous group, and an account of the remainder of the clinical lectures and special demonstrations during the medical home coming week at the University of Pennsylvania last spring. The lectures on diagnosis include a contribution to the study of the ideographic cerebral center, by H. V. Würdemann, Seattle; the radiation of pain in renal calculus, by L. W. Gorham, Baltimore; and Ehrlich's diazo reaction in chronic tuberculosis, by Nathan P. Levin, Edgewater, Colo. The articles on treatment embrace the treatment of noma and of pneumonia at Saint Vincent's home, by Joseph P. Lopez, Philadelphia; reports on autoserotherapy, by C. K. Austin, Paris; demonstration of Uuna's paste in the treatment of leg ulcer, by B. A. Thomas, Philadelphia; treatment of advanced and acute cases of tuberculosis of the lungs, by Joseph Walsh, Philadelphia; observations on the salt free diet and chloride metabolism, by Edward H. Goodman, Philadelphia; treatment of senile gangrene, by superheated air, by G. Dieulafoy, Paris; present status of bacteria therapy, by B. A. Thomas, Philadelphia.

The titles relating to the teeth and oral cavity are, the philosophy of lancing teeth, by Joseph Head, Philadelphia; pyorrhea alveolaris, by Arthur H. Merritt, New York; and oral prophylaxis, by Lucius W. Johnson, U. S. Navy. In gynecology we find considered the causes and treatment of various types of persistent hemorrhage from the uterus, by Brooke M. Anspach, Philadelphia; rare and anomalous conditions, obstetrical and gynecological, by Chauncey D. Palmer, Cincinnati; appendicitis as a complication of pregnancy, etc., by Palmer Findley, Omaha; rupture of ovarian cystomata, by Charles Greene Cumston, Boston.

In medicine the subjects presented are uncinariasis, by M. Howard Fussell, Philadelphia; hydrophobia, by Milton K. Meyers, Philadelphia; aim of the trachoma institute, by Aaron Brad, Philadelphia; and dilatation of the subclavian artery, by Frank G Wilson, Louisville. Surgery offers the surgical treatment of the pancreas, by Charles G. Cumston, Boston; treatment of fracture of the femur of the new born, by P. Samuel Stout, Philadelphia; tuberculous abscess of the hip,—arthritis deformans of the hip, by John L. Porter, Chicago; congenital club foot—punctured wound of the thumb, by J. Garland Sherrill, Louisville; action of bacteria in the peritoneal cavity by George P. Müller, Philadelphia; syphilitic stricture of the rectum, by Bernard Asman, Louisville. A number of miscellaneous titles follow, and then the lectures pertaining to home coming week, edited by John G. Clark. The volume is full of interest and adds to the value of the series.

